# Parental Knowledge, Attitudes and Practices Regarding the Prevention and Home Management of Bronchiolitis in Infants: A Cross-Sectional Study

**DOI:** 10.3390/pediatric18040092

**Published:** 2026-07-08

**Authors:** Melania Vázquez-Ortega, Héctor González-de la Torre, María-Naira Hernández-De Luis, Sergio Mies-Padilla, Claudio-Alberto Rodríguez-Suárez

**Affiliations:** 1 Las Remudas Primary Health Care Centre, Canary Health Service, 35213 Las Palmas de Gran Canaria, Spain; vazquezortega.m@gmail.com (M.V.-O.); nairahernandez@celp.es (M.-N.H.-D.L.); 2 Nursing Department, Faculty of Healthcare Science, Universidad de Las Palmas de Gran Canaria (ULPGC), 35016 Las Palmas de Gran Canaria, Spain; hector.gonzalez@ulpgc.es (H.G.-d.l.T.); claudioalberto.rodriguez@ulpgc.es (C.-A.R.-S.); 3 Research Support Unit, Insular Maternal and Child University Hospital Complex, Canary Health Service, 35016 Las Palmas de Gran Canaria, Spain; 4 Faculty of Healthcare Science, University of Atlántico Medio (UNAM), 35017 Las Palmas de Gran Canaria, Spain

**Keywords:** bronchiolitis, health knowledge, attitudes, practice, primary prevention, infant, parents

## Abstract

Background/Objectives: Acute bronchiolitis is one of the leading respiratory infections in infants and represents a substantial burden on healthcare services. Parents’ knowledge, attitudes and practices are key to its prevention and home management. The aim of this study was to analyze parents’ knowledge, attitudes, and practices regarding the prevention and home management of bronchiolitis in infants in Gran Canaria, Spain. Methods: A cross-sectional observational study was conducted. The Bronchiolitis Knowledge, Attitudes and Practices Questionnaire was used, comprising 26 items grouped into four dimensions: risk factors, signs and symptoms, prevention, and care/pharmacological support. Data were collected using an online questionnaire. Descriptive analyses, nonparametric tests, and multiple linear regression were performed. Statistical analysis was conducted using Jamovi (version 2.4.12). Statistical significance was set at *p* < 0.05. Results: A total of 162 parents were included. The mean normalized total score indicated an overall level of parental knowledge, attitudes, and practices regarding acute bronchiolitis. Prevention was the dimension with the lowest scores, whereas signs and symptoms and care/pharmacological support showed comparatively better results. Higher overall scores were associated with educational level and previous experience with bronchiolitis. Conclusions: Parents showed insufficient knowledge, attitudes and practices, particularly in relation to prevention. Targeted educational interventions are needed to improve the home management of bronchiolitis and help reduce healthcare burden.

## 1. Introduction

Acute bronchiolitis (AB) is a seasonal respiratory infection with a high prevalence in infants, with peak incidence occurring between November and March [1]. It primarily affects children under two years of age and represents a major challenge for healthcare systems due to the substantial burden placed on emergency departments and hospital services. Although its etiology is diverse, morbidity increases when the causative agent is respiratory syncytial virus (RSV), which commonly presents with wheezing, respiratory distress, cough, and fever. Approximately 1% to 3% of cases develop complications such as feeding difficulties, apnea, or inability to maintain adequate oxygen saturation (SpO_2_), resulting in hospitalization and increased healthcare demand [1].

Over the past three decades, hospitalizations related to AB have been frequent and have generated a considerable economic and healthcare burden for both health systems and families. However, during the COVID-19 pandemic in Spain, pediatric hospital admissions due to bronchiolitis decreased by 94.1% compared with the pre-pandemic period, accompanied by a reduction in cases and changes in seasonal patterns, including earlier peaks and redistribution of cases. Before the pandemic, RSV accounted for most AB cases (up to 73% between November and February), whereas during the pandemic respiratory infections declined to approximately 10%, with SARS-CoV-2 and rhinovirus becoming more prominent pathogens [2]. More recently, the implementation of maternal RSV vaccination and passive immunization with monoclonal antibodies, particularly nirsevimab, has further contributed to reducing the burden of RSV-associated disease and hospitalizations in infants, representing an important advance in AB prevention [3].

Although clinical practice guidelines for the diagnosis, treatment, and follow-up of AB are available, variability in their implementation and discrepancies among recommendations persist [4]. Pharmacological treatments such as bronchodilators, corticosteroids, and antivirals have been proposed; however, evidence demonstrates limited benefits and marked heterogeneity in their clinical use, contributing to variability in healthcare practice [5]. In this context, the management of AB should include not only clinical treatment but also health education directed at parents and caregivers. Given the high frequency of AB during the first years of life and the absence of specific pharmacological treatments for most cases, parental education plays a fundamental role in ensuring appropriate home management, promoting early recognition of warning signs, and supporting the appropriate use of healthcare services.

Regarding home care, recommendations include avoiding exposure to tobacco smoke and other harmful substances, maintaining adequate hydration, performing nasal saline irrigation when necessary, and positioning the infant in a semi-upright position. Furthermore, educating families about the disease and the recognition of warning signs requiring urgent medical attention is essential [6]. These warning signs include apnea, perioral or distal cyanosis, tachypnea, respiratory distress, feeding refusal, vomiting, and general deterioration.

Preventive measures primarily include hand hygiene, avoiding close contact with symptomatic individuals, and educating families about strategies to reduce viral transmission. In recent years, maternal RSV vaccination during pregnancy and passive immunization wit monoclonal antibodies has expanded the preventive strategies available to reduce RSV-associated disease and hospitalizations in infants [4]. Nevertheless, parental knowledge and adherence to recommended preventive measures remain fundamental for reducing AB transmission and supporting appropriate home management [4,6].

Although previous studies have explored parental knowledge regarding childhood respiratory infections and antibiotic use, evidence specifically addressing knowledge, attitudes, and practices related to AB remains limited, particularly in Spain and using validated disease-specific instruments. Moreover, little is known about parents’ preparedness to recognize warning signs, implement preventive measures, and appropriately manage bronchiolitis at home. Identifying these knowledge gaps is essential for designing targeted educational interventions aimed at improving home management, promoting appropriate healthcare utilization, and ultimately reducing the burden of AB on healthcare services. Therefore, the aim of this study was to analyze parental knowledge, attitudes, and practices regarding the prevention and home management of AB in infants in Gran Canaria, Spain.

## 2. Materials and Methods

### 2.1. Design

A cross-sectional descriptive observational study with an analytical component was conducted.

### 2.2. Participants and Sample

The target population consisted of parents of infants aged ≤2 years residing in Gran Canaria (Las Palmas, Spain) during 2024. A non-probabilistic convenience sampling method was employed. An a priori sample size of 385 participants was estimated to provide adequate precision for the descriptive analyses planned in this cross-sectional study, assuming a 95% confidence level, a precision of ±0.05, and a standard deviation of 0.5. A loss-to-follow-up rate was not considered due to the cross-sectional design and single-time-point data collection.

### 2.3. Inclusion and Exclusion Criteria

Parents of infants aged 0–24 months were included if they met at least one of the following criteria: (1) the infant had a history of AB, or (2) the infant had no history of AB but had at least one older sibling with a previous episode of AB. Parents who did not understand or speak Spanish, as well as incomplete questionnaires, were excluded.

### 2.4. Variables and Instrument

Data were collected on parental sex (male, female, prefer not to answer), infant sex (boy, girl, prefer not to answer), parental age (years), infant age (months), educational level (no formal education, primary, secondary, vocational training, university), number of children, history of AB in older siblings (yes/no), history of AB in the current infant (yes/no), and hospitalization due to AB (yes/no).

The “Parental Knowledge, Attitudes, and Practices on Bronchiolitis Prevention and Home Management Questionnaire” was used for assessment purposes. The instrument was developed and validated in the Spanish population [7]. It consists of 26 items grouped into four dimensions: risk factors (items 1–5), signs and symptoms (items 6–13), prevention (items 14–18), and care/pharmacological support (items 19–26). Responses were recorded using a 5-point Likert scale (1 = strongly disagree; 5 = strongly agree). Items 22–25 were negatively worded and were reverse-coded before calculating total and dimension scores so that higher values indicated better knowledge, attitudes, and practices. Item 26 was excluded from the total score in accordance with the original instrument. The total score ranged from 26 to 130 points. A normalized total score was calculated following the authors’ method: items 1–21 were scored from −2 to +2, items 22–25 were reverse-coded, and item 26 was excluded. Thus, the global score ranged from −50 to +50, with positive values indicating better knowledge, attitudes, and practices [7].

### 2.5. Dimension Score Standardization

To facilitate comparisons across dimensions with different score ranges, mean dimension scores were standardized using the min–max scaling method according to the following formula where *X* is the observed score, *X_min_* is the minimum possible score, *X_max_* is the maximum possible score, and *X_norm_* is the standardized score:

*X_norm_* = (*X* − *X_min_*)/(*X_max_* − *X_min_*)

Standardized scores ranged from 0 to 1 and were categorized into five levels: very low (0.00–0.20), low (0.21–0.40), moderate (0.41–0.60), high (0.61–0.80), and very high (0.81–1.00).

### 2.6. Data Collection

Data were collected using an anonymous and voluntary online questionnaire administered through Google Forms^®^ (Google LLC, Mountain View, CA, USA). The survey was distributed via email and QR codes. The link included an information sheet, study objectives, and informed consent. Before accessing the questionnaire, eligible participants were provided with an information sheet describing the objectives of the study and were required to provide electronic informed consent before completing the survey. Only after consent was provided could the questionnaire be completed. Data collection took place between May and June 2024.

### 2.7. Data Analysis

Qualitative variables were described using absolute frequencies and percentages, whereas quantitative variables were summarized using mean (M), standard deviation (SD), and 95% confidence intervals (95% CI). Normality was assessed using the Shapiro–Wilk test.

Given the distribution of the data, nonparametric tests were applied, including the Mann–Whitney U test, Kruskal–Wallis test (*χ*^2^), and Spearman’s correlation coefficient (*ρ*). Within-subject comparisons across questionnaire dimensions were performed using the Friedman test, followed by Durbin–Conover post hoc comparisons with Bonferroni correction. Additional post hoc analyses were conducted using the Dwass–Steel–Critchlow–Fligner (DSCF) test when appropriate.

Effect sizes were calculated using rank-biserial correlation and epsilon squared (*ε*^2^). Correlation strength was interpreted as very weak (<0.20), weak (0.20–0.39), moderate (0.40–0.59), strong (0.60–0.79), and very strong (≥0.80), taking into account the direction of the association. Internal consistency was assessed using Cronbach’s alpha (*α*) and McDonald’s omega (*ω*).

Multiple linear regression analysis (enter method) was performed to identify factors associated with the total score. Model assumptions were verified, and unstandardized coefficients (*β*), standard errors, 95% CI, and standardized coefficients were reported. Statistical significance was established at *p* < 0.05. All analyses were conducted using Jamovi software (version 2.4.12; The Jamovi Project, Sydney, Australia).

### 2.8. Ethical Considerations

Authorization to use the instrument was obtained from the original authors. Confidentiality was guaranteed in accordance with current regulations (Organic Law 3/2018 and Regulation (EU) 2016/679). The study followed the principles of the Declaration of Helsinki and the Belmont Report. Electronic informed consent was obtained from all participants before data collection. The protocol was approved by the Research Ethics Committee of the province of Las Palmas (code: 2024-201-1).

## 3. Results

A total of 173 responses were obtained, of which 9 were excluded for not meeting the inclusion criteria (infants aged >2 years), resulting in a final sample of *n* = 162 parents (42.08%). The instrument demonstrated adequate internal consistency, with Cronbach’s alpha (*α* = 0.787) and McDonald’s omega (*ω* = 0.841). Table 1 presents the sociodemographic and clinical characteristics of the sample.

The mean total score was 52.16 (SD = 10.12). The mean score for the risk factors dimension was 12.20 (SD = 3.14), whereas the signs and symptoms dimension showed a mean of 15.12 (SD = 4.35). The prevention dimension had a mean score of 9.17 (SD = 3.21), while the care and pharmacological support dimension showed a mean of 15.68 (SD = 4.62).

The lowest standardized scores were observed in the prevention (M = 0.21) and signs and symptoms (M = 0.22) dimensions, whereas the risk factors (M = 0.36) and care and pharmacological support (M = 0.33) dimensions showed relatively higher values. Nevertheless, the overall level of knowledge remained low.

The mean normalized total score was −22.84 (SD = 10.12; 95% CI: −24.40 to −21.28). These findings indicate that the mean score was below the cutoff point established for the instrument, reflecting an overall low level of parental knowledge, attitudes, and practices regarding the prevention and home management of AB in infants. Table 2 presents the descriptive statistics for the questionnaire scores.

To analyze differences between questionnaire dimensions, the nonparametric Friedman test was performed, revealing statistically significant differences among the dimensions (*p* < 0.001). Post hoc comparisons using the Durbin–Conover test with Bonferroni correction showed significant differences between most dimensions (*p* < 0.001).

Specifically, the prevention dimension showed significantly lower scores than the risk factors, signs and symptoms, and care and pharmacological support dimensions (all *p* < 0.001). In addition, significant differences were observed between the risk factors dimension and the remaining dimensions (*p* < 0.001).

No statistically significant differences were found between the signs and symptoms and care and pharmacological support dimensions (*p* = 0.588). Table 3 presents the comparisons between questionnaire dimensions.

In the bivariate analysis, no significant differences were found in any questionnaire dimension or in the total score according to infant sex. Similarly, no differences were observed between participants with previous children with AB, a history of AB in the current infant, or prior hospitalizations due to AB. No statistically significant differences were identified according to parental educational level.

In contrast, statistically significant differences were observed in the total questionnaire score according to parent sex (*p* = 0.037; *ε*^2^ = 0.041), with higher scores among men. Post hoc analysis indicated that these differences were specifically observed between female and male (*p* = 0.033).

Regarding infant sex, although statistically significant differences were found in the prevention dimension (*p* = 0.010; *ε*^2^ = 0.058), post hoc comparisons did not reach statistical significance, suggesting the absence of clear differences between groups, as shown in Table 4.

No statistically significant correlations were observed between parent age, infant age, or number of children and the total questionnaire score or most of its dimensions. However, several significant associations were identified. Infant age showed a weak negative correlation with the signs and symptoms dimension (*ρ* = −0.158; *p* = 0.045) and with the care and pharmacological support dimension (*ρ* = −0.171; *p* = 0.030), indicating that older infant age was associated with lower scores in these dimensions.

The number of children showed a weak positive correlation with parent age (*ρ* = 0.161; *p* = 0.042), with no significant associations observed for the remaining variables.

Regarding the questionnaire dimensions, positive correlations were found between several dimensions. The risk factors dimension showed weak positive correlations with signs and symptoms (*ρ* = 0.229; *p* = 0.003) and prevention (*ρ* = 0.210; *p* = 0.007). The signs and symptoms dimension showed a moderate positive correlation with prevention (*ρ* = 0.449; *p* < 0.001) and a weak positive correlation with care and pharmacological support (*ρ* = 0.233; *p* = 0.003). Prevention also showed a weak positive correlation with care and pharmacological support (*ρ* = 0.208; *p* = 0.008).

Finally, the total questionnaire score showed moderate-to-strong positive correlations with all dimensions, supporting the internal consistency of the instrument. Table 5 presents the correlation analyses.

The linear regression model was statistically significant (*F* = 1.83; *p* = 0.040), explaining 15.1% of the variance (*R*^2^ = 0.151). Significant associations were identified for educational level (university education vs. vocational training: *β* = −4.96; *p* = 0.023), having previous children with bronchiolitis (*β* = 4.24; *p* = 0.043), and a history of bronchiolitis in the current child (*β* = −4.61; *p* = 0.012). No statistically significant associations were observed for the remaining variables included in the model. These results are presented in Table 6.

## 4. Discussion

The results of this study showed that the overall level of parental knowledge, attitudes, and practices regarding AB was low, which is consistent with previous research identifying important gaps in health education in the field of pediatric respiratory infections [8,9]. This finding is particularly relevant considering the key role parents play in the home management of this condition.

From a sociodemographic perspective, a higher participation of women was observed, in line with previous literature conducted in similar contexts. Furthermore, parents, especially during a first episode of AB, have been reported to experience anxiety and feel unprepared, although they are generally able to recognize some clinical signs and identify when medical attention is required [10]. These findings are consistent with the results of the present study.

The study population was predominantly composed of mothers, and most participants had completed vocational or secondary education. In addition, the majority of families had one or two children, and approximately one-third had previous experience with AB. These characteristics are consistent with the profile of parents commonly involved in the day-to-day care of infants in primary healthcare settings and should be considered when interpreting the findings. Although the predominance of mothers may have influenced the perspectives captured, it also reflects the parent most frequently responsible for seeking healthcare and providing home care during childhood illnesses.

The dimensional analysis revealed a heterogeneous profile, with prevention and recognition of signs and symptoms representing the lowest-scoring areas. This pattern suggests difficulties in the early identification of risk situations and in the adoption of preventive measures, both of which are essential to reducing the transmission and complications of bronchiolitis. Similar findings have been described in previous studies, where parents demonstrated greater knowledge regarding disease management after symptom onset than regarding preventive strategies [11]. In this regard, the low scores observed in the prevention dimension highlight the need for interventions focused on measures such as hand hygiene, limiting close contact, and improving ventilation, all of which are widely recommended in clinical guidelines [4].

Several studies have also reported substantial exposure to modifiable risk factors, such as passive smoking [12]. Evidence indicates that prenatal and postnatal tobacco exposure significantly increases the risk of AB during the first years of life, with nearly half of cases potentially attributable to maternal smoking [13]. These findings reinforce the importance of incorporating specific preventive content into educational interventions aimed at parents.

Regarding the recognition of signs and symptoms, the findings suggest that parents experience difficulties identifying warning signs such as respiratory distress or cyanosis, which may delay seeking medical care. This issue is particularly relevant because early recognition is associated with improved clinical outcomes [4]. In addition, the limited knowledge observed regarding fever management is consistent with previous studies describing misconceptions about fever among the general population [14].

Although the care and pharmacological support dimension showed relatively higher scores, potentially inappropriate practices were identified, including the use of antibiotics or bronchodilators without medical prescription. This finding is consistent with the literature describing inappropriate antibiotic use in viral respiratory infections, often associated with insufficient knowledge and misconceptions [8,15]. Moreover, this situation may be influenced by variability in clinical practice and parental perceptions regarding the necessity of treatment [16].

Overall knowledge levels in this study were lower than those reported in other contexts. For example, a multicenter study found that up to 71% of parents reported having a basic or good level of knowledge about RSV, particularly those with previous experience [17]. This discrepancy may be explained by contextual or methodological differences, or by a weaker conceptual association between AB and RSV among participants in the present study.

Previous research has also shown that many parents feel poorly informed, have difficulties recognizing severe symptoms, and tend to overestimate the effectiveness of antibiotics in respiratory infections [18]. These findings further emphasize the need to strengthen health education initiatives.

Regarding clinical factors, evidence suggests that variables such as infant age, male sex, family smoking, and the presence of older siblings may influence the clinical course and healthcare burden of bronchiolitis [19]. However, in the present study, sociodemographic variables showed limited influence, suggesting that educational needs may be widespread across the population rather than concentrated in specific groups.

The multivariate analysis showed that the regression model explained a relatively small proportion of the variance, indicating that parental knowledge, attitudes, and practices regarding AB are influenced by multiple determinants beyond those included in the present study. In addition to health literacy and the quality of information received, factors such as socioeconomic status, access to reliable healthcare information, previous educational interventions provided by healthcare professionals, and parents’ ability to seek and appraise digital health information may also influence parental preparedness and decision-making. These variables were not assessed in the present study and may account for part of the unexplained variance. Future studies should incorporate these determinants into predictive models to achieve a more comprehensive understanding of parental educational needs and to guide the development of more effective educational interventions. 

Interestingly, previous experience with AB in older children was associated with higher scores, suggesting a learning effect whereby parents acquire knowledge and confidence through previous disease episodes and interactions with healthcare professionals. In contrast, the negative association observed among parents whose current infant had experienced AB may reflect the uncertainty, emotional stress, and perceived complexity associated with managing an acute illness episode. Parents facing AB in their current infant may also become more aware of the challenges involved, leading to a more critical self-assessment of their own knowledge and practices. This interpretation is consistent with previous studies showing that acute pediatric illnesses may increase parental anxiety and reduce self-confidence despite previous exposure to health information [20].

In this context, the role of healthcare professionals, particularly pediatric nurses, is crucial. In both primary care and hospital settings, nurses play an essential role in health education by facilitating the acquisition of knowledge and practical skills among parents. Evidence suggests that nurse-led educational interventions improve caregivers’ ability to recognize warning signs, optimize home management, and reduce the inappropriate use of healthcare resources [4,20]. Furthermore, continuity of care between healthcare levels may support the consolidation of these skills, contributing to safer and more effective home management.

Overall, these findings highlight the need to strengthen health education interventions aimed at parents, particularly in the areas of prevention and early recognition of warning signs. Such interventions should focus on providing clear, practical, and evidence-based information to promote the appropriate use of healthcare resources and reduce potentially inappropriate practices.

Among the main strengths of this study is the use of a specific instrument validated in the Spanish population, as well as the dimensional analysis, which enabled the identification of specific areas requiring improvement. In addition, the combination of bivariate and multivariate analyses strengthens the interpretation of the findings.

However, several limitations should be acknowledged. The cross-sectional design precludes establishing causal relationships. Furthermore, the final sample size was lower than initially estimated, which may have reduced the statistical power to detect small associations and limited the precision of the estimated effects, thereby increasing the risk of type II error. The use of non-probability convenience sampling may also have introduced selection bias and limits the representativeness of the sample, reducing the generalizability of the findings. In addition, data were collected through a self-administered online questionnaire, which may have preferentially recruited parents with greater digital literacy, internet access, or engagement with healthcare information, potentially underrepresenting more vulnerable populations with lower digital skills. This recruitment strategy may therefore have introduced additional selection bias. Finally, the limited explanatory capacity of the multivariable model suggests that other relevant factors, such as health literacy, socioeconomic status, access to reliable healthcare information, and previous educational interventions provided by healthcare professionals, may also influence parental knowledge, attitudes, and practices and should be considered in future research.

## 5. Conclusions

Parents of infants showed an overall low level of knowledge, attitudes, and practices regarding the prevention and home management of AB, with mean scores falling below the threshold established by the instrument. A heterogeneous profile across dimensions was identified, with prevention emerging as the area with the greatest deficiencies, highlighting the need to strengthen educational interventions in this domain. In contrast, the dimensions related to the recognition of signs and symptoms and home care showed better results, possibly associated with parents’ previous experience with illness episodes.

No relevant differences were observed according to most sociodemographic and clinical variables, suggesting that educational needs are widespread across the population. However, factors such as educational level and prior experience with AB were associated with overall scores, indicating potential profiles of greater or lesser vulnerability.

Overall, these findings underscore the importance of designing and implementing targeted health education strategies for parents, particularly those focused on prevention and the appropriate use of therapeutic measures, in order to improve the home management of AB and reduce the burden on healthcare services.

Future research should evaluate the effectiveness of targeted educational interventions and their impact on improving parental knowledge, as well as on reducing unnecessary healthcare visits and hospitalizations.

## Figures and Tables

**Table 1:** Sociodemographic and Clinical Characteristics of the Sample.

Variables		Mean (SD)	95% CI	*n* (%)
Parent age (years)		32.13 (10.29)	30.53 to 33.73	
Infant age (months)		9.67 (6.81)	8.62 to 10.73	
Number of children		1.58 (0.81)	1.46 to 1.71	
Parent sex	Female			121 (74.7)
	Male			40 (24.7)
	Prefer not to answer			1 (0.6)
Infant sex	Girl			73 (45.1)
	Boy			85 (52.5)
	Prefer not to answer			4 (2.5)
Educational level	No formal education			6 (3.7)
	Primary education			20 (12.3)
	Secondary education			43 (26.5)
	Vocational training			60 (37.0)
	University education			33 (20.4)
History of bronchiolitis in older siblings	No			119 (73.5)
	Yes			43 (26.5)
Bronchiolitis in the current infant	No			102 (63.0)
	Yes			60 (37.0)
Hospitalization due to bronchiolitis	No			118 (72.8)
	Yes			44 (27.2)

**Table 2:** Descriptive Results of the Parental Knowledge, Attitudes, and Practices Questionnaire on Bronchiolitis Prevention and Home Management.

Items	Mean (SD)	95% CI
Dimension 1. Risk factors	12.20 (3.14)	11.71 to 12.68
1. I consider exposure to tobacco smoke a risk factor for bronchiolitis	1.44 (0.69)	1.33 to 1.55
2. I consider lack of breastfeeding a risk factor for bronchiolitis	2.91 (1.19)	2.73 to 3.10
3. I consider that living with siblings may affect my child’s respiratory health	3.59 (1.24)	3.40 to 3.79
4. I consider that attending daycare may influence my child’s respiratory health	2.73 (1.21)	2.55 to 2.92
5. I consider it important to seek information from health sources to manage a respiratory infection	1.52 (0.71)	1.41 to 1.63
Dimension 2. Signs and symptoms	15.12 (4.35)	14.44 to 15.79
6. I believe I could identify nasal flaring if it appeared	2.20 (0.92)	2.05 to 2.34
7. I believe I could identify chest retractions	2.19 (1.01)	2.03 to 2.34
8. I believe I could identify cyanosis (bluish lips or nails due to lack of oxygen)	2.01 (0.90)	1.87 to 2.15
9. I believe I could identify lethargy or drowsiness in my child	1.82 (0.76)	1.70 to 1.94
10. I believe I could identify breathing difficulty in my child	1.71 (0.71)	1.60 to 1.82
11. I believe I could recognize when food and fluid intake is concerning	2.11 (0.87)	1.97 to 2.25
12. I know the temperature threshold for fever in young children	1.75 (0.77)	1.63 to 1.82
13. If any warning signs appear, I would seek medical care for my child	1.33 (0.62)	1.23 to 1.42
Dimension 3. Prevention	9.17 (3.21)	8.67 to 9.66
14. I consider handwashing effective in preventing virus transmission	1.63 (0.82)	1.50 to 1.76
15. I consider covering coughs/sneezes effective in preventing transmission	1.73 (0.83)	1.60 to 1.86
16. I consider limiting visits effective in preventing transmission	2.12 (0.96)	1.97 to 2.27
17. I consider avoiding crowds effective in preventing transmission	2.01 (0.93)	1.87 to 2.16
18. I consider ventilation effective in preventing transmission	1.68 (0.75)	1.56 to 1.79
Dimension 4. Care and pharmacological support	15.68 (4.62)	14.96 to 16.39
19. I monitor fluid intake during my child’s respiratory infection	1.77 (0.76)	1.65 to 1.89
20. I adjust feeding during my child’s respiratory infection	1.77 (0.74)	1.65 to 1.88
21. I perform nasal irrigation during respiratory infection	1.73 (0.71)	1.62 to 1.85
22. I would use antipyretics without fever in bronchiolitis	3.07 (1.36)	2.86 to 3.28
23. I would use antibiotics without prescription	3.90 (1.38)	3.69 to 4.12
24. I would use bronchodilators without medical supervision	3.74 (1.42)	3.52 to 3.96
25. I use mucolytics and antitussives for respiratory infections	2.88 (1.26)	2.69 to 3.08
Total score (non-normalized)	52.16 (10.12)	50.59 to 53.73
Total score (normalized)	−22.84 (10.12)	−24.40 to 21.28
26. I have taken my child to respiratory physiotherapy sessions	3.02 (1.32)	2.82 to 3.23

**Table 3:** Comparison Between Questionnaire Dimensions.

Dimension	Mean	Post Hoc Comparisons (*p* Value)
Risk factors	12.20	vs. Signs and symptoms (<0.001) vs. Prevention (<0.001) vs. Care and pharmacological support (<0.001)
Signs and symptoms	15.12	vs. Prevention (<0.001) vs. Care and pharmacological support (0.588)
Prevention	9.17	vs. Care and pharmacological support (<0.001)
Care and pharmacological support	15.68	—

**Table 4:** Significant Results of the Bivariate Analysis.

Variable	Dimension	Group	Mean (SD)	*p*	Effect Size	Post Hoc (DSCF)
Parent sex	Total score	Female (*n* = 121)	51.43 (10.419)	0.037	ε^2^ = 0.041	Female vs. Male (*p* = 0.033)
		Male (*n* = 40)	54.27 (9.08)			
		Prefer not to answer (*n* = 1)	56.0 (0.0)			
Infant sex	Prevention	Boys (*n* = 73)	9.82 (3.20)	0.010	ε^2^ = 0.058	Girls vs. Boys (*p* = 0.071) Prefer not to answer vs. Boys (*p* = 0.051) Prefer not to answer vs. Girls (*p* = 0.144)
		Girls (*n* = 85)	8.75 (3.13)			
		Prefer not to answer (*n* = 4)	6.0 (2.0)			

*ε*^2^ = epsilon squared; DSCF = Dwass–Steel–Critchlow–Fligner; *p* < 0.05.

**Table 5:** Correlations Between Quantitative Variables (Spearman’s *ρ*).

Variables	1	2	3	4	5	6	7	8
Parent age (1)	—							
Infant age (2)	0.115 (*p* = 0.144)	—						
Number of children (3)	0.161 (*p* = 0.042) *	0.029 (*p* = 0.713)	—					
Risk factors (4)	−0.145 (*p* = 0.066)	0.124 (*p* = 0.115)	−0.026 (*p* = 0.744)	—				
Signs and symptoms (5)	−0.073 (*p* = 0.353)	−0.158 (*p* = 0.045) *	−0.043 (*p* = 0.586)	0.229 (*p* = 0.003) **	—			
Prevention (6)	−0.037 (*p* = 0.645)	0.025 (*p* = 0.754)	0.137 (*p* = 0.086)	0.210 (*p* = 0.007) **	0.449 (*p* < 0.001) ***	—		
Care and pharmacological support (7)	−0.103 (*p* = 0.192)	−0.171 (*p* = 0.030) *	0.029 (*p* = 0.714)	−0.010 (*p* = 0.898)	0.233 (*p* = 0.003) **	0.208 (*p* = 0.008) **	—	
Total score (8)	−0.135 (*p* = 0.087)	−0.075 (*p* = 0.341)	0.018 (*p* = 0.825)	0.433 (*p* < 0.001) ***	0.723 (*p* < 0.001) ***	0.685 (*p* < 0.001) ***	0.631 (*p* < 0.001) ***	—

*ρ* = Spearman’s correlation coefficient; *p* < 0.05 *, *p* < 0.01 **, *p* < 0.001 ***.

**Table 6:** Linear Regression Model for the Total Scale Score.

Predictor	*β* (SE)	95% CI	*p*	Standardized *β*
Constant	58.27 (3.20)	51.94 to 64.60	<0.001	—
Parent age (years)	−0.09 (0.08)	−0.24 to 0.07	0.275	−0.09
Infant age (months)	−0.14 (0.13)	−0.39 to 0.11	0.271	−0.09
Number of children	0.00 (1.09)	−2.15 to 2.16	0.998	0.00
Parent sex				
Male vs. Female	2.54 (1.84)	−1.10 to 6.18	0.170	0.25
Prefer not to answer vs. Women	0.64 (10.17)	−19.46 to 20.74	0.950	0.06
Infant sex				
Girls vs. Child	−2.94 (1.60)	−6.10 to 0.22	0.068	−0.29
Prefer not to answer vs. Child	−7.55 (5.08)	−17.58 to 2.48	0.139	−0.76
Educational level				
Secondary vs. Vocational training	1.19 (2.07)	−2.91 to 5.29	0.567	0.12
University vs. Vocational training	−4.96 (2.16)	−9.23 to −0.69	0.023 *	−0.50
Primary vs. Vocational training	1.26 (2.53)	−3.74 to 6.26	0.618	0.13
No formal education vs. Vocational training	5.33 (4.78)	−4.12 to 14.78	0.267	0.53
Previous children with bronchiolitis (Yes vs. No)	4.24 (2.07)	0.14 to 8.33	0.043 *	0.42
Bronchiolitis in current child (Yes vs. No)	−4.61 (1.81)	−8.19 to −1.03	0.012 *	−0.46
Hospitalization (Yes vs. No)	0.81 (1.89)	−2.92 to 4.55	0.667	0.08

Overall model: *R* = 0.388; *R*^2^ = 0.151; *F* = 1.83; *p* = 0.040. *β* = unstandardized coefficient; SE = standard error; CI = confidence interval; * Statistically significant (*p* < 0.05).

## Data Availability

The data used in this research are confidential and are protected in a coded and anonymized database kept by the research group in accordance with Spanish regulations. However, raw data concerning the preference scores for the thematic areas can be shared with those researchers who contact the corresponding author if requested with a reasoned and logical request.

## Abbreviations

The following abbreviations are used in this manuscript:

AB	Acute Bronchiolitis
QR	Quick Response
RSV	Respiratory Syncytial Virus
SpO_2_	Peripheral Oxygen Saturation

## References

[1] Caballero M.T., Polack F.P., Stein R.T. (2017). Viral bronchiolitis in young infants: New perspectives for management and treatment. J. Pediatr..

[2] Rius-Peris J.M., Lucas-García J., García-Peris M., Escrivá Tomás P., Sequí-Canet J.M., González de Dios J. (2021). Pandemia por COVID-19 y su repercusión sobre las hospitalizaciones por bronquiolitis en el Centro y Este de España. An. Pediatr..

[3] Ralston S.L., Lieberthal A.S., Meissner H.C., Alverson B.K., Baley J.E., Gadomski A.M., Johnson D.W., Light M.J., Maraqa N.F., Mendonca E.A. (2014). Clinical practice guideline: The diagnosis, management, and prevention of bronchiolitis. Pediatrics.

[4] Kirolos A., Manti S., Blacow R., Tse G., Wilson T., Lister M., Cunningham S., Campbell A., Nair H., Reeves R.M. (2020). A systematic review of clinical practice guidelines for the diagnosis and management of bronchiolitis. J. Infect. Dis..

[5] National Institute for Health and Care Excellence (NICE) (2021). Bronchiolitis in Children: Diagnosis and Management.

[6] Francisco L., Cruz-Cañete M., Pérez C., Couceiro J.A., Otheo E., Launes C., Rodrigo C., Jiménez A.B., Llorente M., Montesdeoca A. (2023). Nirsevimab for the prevention of respiratory syncytial virus disease in children. An. Pediatr. (Engl. Ed.).

[7] Delgado-Castillejo E., Carratalá-Tejada M., Molina-Rueda F. (2023). Design and reliability study of a parental knowledge, attitude, and practice questionnaire on prevention and management of acute bronchiolitis of children under two years of age. An. Sist. Sanit. Navar..

[8] Cantarero-Arévalo L., Hallas M.P., Hansen E.H. (2017). Parental knowledge of antibiotic use in children with respiratory infections: A systematic review. Int. J. Pharm. Pract..

[9] Al Hashmi A.S., Al Zakwani I., Al Mahruqi G., Al Maskari N., Al Balushi Z. (2021). Parental knowledge, attitudes and practices regarding antibiotic use for upper respiratory tract infections in children. J. Multidiscip. Healthc..

[10] Campbell A., Hartling L., Louie-Poon S., Scott S.D. (2020). Parent experiences caring for a child with bronchiolitis: A qualitative study. J. Patient Exp..

[11] Albayrak A., Colak D., Albayrak R. (2021). Evaluation of parental knowledge, attitudes and practices regarding antibiotic use in acute upper respiratory tract infections in children. BMC Pediatr..

[12] Friedman J.N., Rieder M.J., Walton J.M., Canadian Paediatric Society, Acute Care Committee, Drug Therapy and Hazardous Substances Committee (2014). Bronchiolitis: Recommendations for diagnosis, monitoring and management of children one to 24 months. Paediatr. Child Health.

[13] Bermúdez Barrezueta L., Miñambres Rodríguez M., Palomares Cardador M., Torres Ballester I., López Casillas P., Moreno Carrasco J., Pino Vázquez A. (2021). Efecto de la exposición prenatal y posnatal al tabaco en el desarrollo de bronquiolitis aguda durante los dos primeros años de vida. An. Pediatr..

[14] Chiappini E., Parretti A., Becherucci P., Pierattelli M., Bonsignori F., Galli L., de Martino M. (2012). Parental and medical knowledge and management of fever in Italian pre-school children. BMC Pediatr..

[15] Hersh A.L., Jackson M.A., Hicks L.A., Brady M.T., Byington C.L., Davies H.D., Edwards K.M., Maldonado Y.A., Murray D.L., Committee on Infectious Diseases (2013). Principles of judicious antibiotic prescribing for upper respiratory tract infections in pediatrics. Pediatrics.

[16] Montejo Fernández M., Benito Manrique I., Montiel Eguía A., Benito Fernández J. (2019). Una iniciativa para reducir el uso de medicación innecesaria en lactantes con bronquiolitis en atención primaria. An. Pediatr..

[17] Lee Mortensen G., Harrod-Lui K. (2022). Parental knowledge about respiratory syncytial virus (RSV) and attitudes to infant immunization with monoclonal antibodies. Expert Rev. Vaccines.

[18] Gates M., Shulhan-Kilroy J., Featherstone R., MacGregor T., Scott S.D., Hartling L. (2019). Parent experiences and information needs related to bronchiolitis: A mixed studies systematic review. Patient Educ. Couns..

[19] Florin T.A., Plint A.C., Zorc J.J. (2017). Viral bronchiolitis. Lancet.

[20] Coyne I., Hallström I., Söderbäck M. (2016). Reframing the focus from a family-centred to a child-centred care approach for children’s healthcare. J. Child Health Care.

